# Letter to the editor regarding the paper “S2k-Guideline hand antisepsis and hand hygiene”

**DOI:** 10.3205/dgkh000537

**Published:** 2025-03-06

**Authors:** Maren Eggers, Katrin Steinhauer, Florin H.H. Brill

**Affiliations:** 1 Labor Prof. Dr. G. Enders MVZ GbR, Stuttgart, Germany; 2 bactologicum GmbH, Itzehoe, Germany; 3 Dr. Brill + Partner GmbH Institute for Hygiene and Microbiology, Hamburg, Germany

## Letter to the editor

Dear editor,

The authors noticed a sensitive mistake in the paper *S2k-Guideline hand antisepsis and hand hygiene* in chapter 5, recommendation 28 [1]. The claims used for activity against viruses are misspelled and may lead to misleading interpretations of the claims. This is likely due to a translation failure from German to English. Consequently, we would like to give the correct claims in Table 1 (Tab. 1).

## Notes

### Competing interests

The author declares that he has no competing interests.

### Funding

None. 

### Authors’ ORCIDs

- Eggers M: https://orcid.org/0000-0001-8485-9485
- Steinhauer K: https://orcid.org/0000-0002-4218-6152
- Brill FHH: https://orcid.org/0000-0001-9681-8752

## Figures and Tables

**Table 1:**
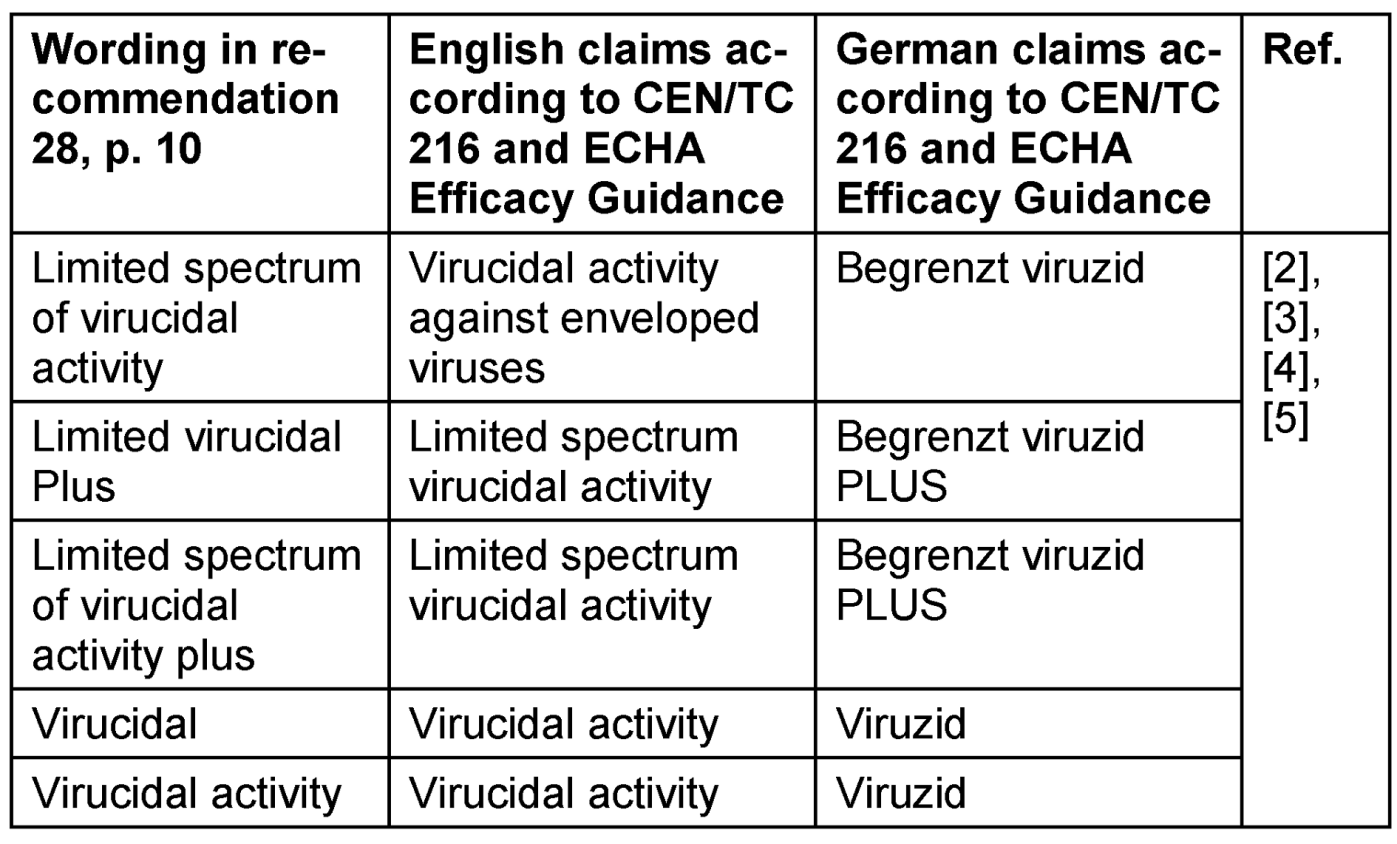
Activity claims for disinfectants and antiseptics against viruses in the human medicine area
